# Strategies to Prevent Underage Drinking

**Published:** 2002

**Authors:** Kelli A. Komro, Traci L. Toomey

**Affiliations:** Kelli A. Komro, M.P.H., Ph.D., and Traci L. Toomey, M.P.H., Ph.D., are assistant professors in the Division of Epidemiology, School of Public Health, University of Minnesota, Minneapolis, Minnesota

**Keywords:** underage drinking, prevention strategy, school-based prevention, curriculum, prevention through alternative activities, skills building, family focused prevention, alcohol or other drug (AOD) public policy strategy, minimum drinking age, availability or accessibility to minors, community-based prevention

## Abstract

Alcohol use by underage drinkers is a persistent public health problem in the United States, and alcohol is the most commonly used drug among adolescents. Accordingly, numerous approaches have been developed and studied that aim to prevent underage drinking. Some approaches are school based, involving curricula targeted at preventing alcohol, tobacco, or marijuana use. Other approaches are extracurricular, offering activities outside of school in the form of social or life skills training or alternative activities. Other strategies strive to involve the adolescents’ families in the prevention programs. Policy strategies also have been implemented that have increased the minimum legal drinking age, reduced the commercial and social access of adolescents to alcohol, and reduced the economic availability of alcohol. Approaches involving the entire community also have been employed. Several programs (e.g., the Midwestern Prevention Project and Project Northland) have combined many of these strategies.

Underage drinking is a persistent public health problem in the United States. Alcohol use initiation rates for children rise quickly from age 10 up to about age 13, when they reach more than 50 percent. Subsequently, initiation rates begin to slow again (Kosterman et al. 2000). Moreover, alcohol is the most commonly used drug among adolescents. For example, among eighth-grade students (who are ages 13 to 14) surveyed in the 1999 national representative sample of the Monitoring the Future study, 52 percent reported having consumed alcohol in their lifetime, and 25 percent reported having been drunk in their lifetime. In addition, 24 percent of the eighth graders reported having used alcohol in the past month and 9 percent reported having been drunk in the past month (Johnston et al. 2000). These rates are higher than those for use of tobacco or any illegal drug (Johnston et al. 2000).

A strong relationship appears to exist between alcohol use among youth and many social, emotional, and behavioral problems, such as using illegal drugs, fighting, stealing, driving under the influence of alcohol and/or other drugs, skipping school, feeling depressed, and deliberately trying to hurt or kill themselves. In addition to the problems that occur during adolescence, early initiation of alcohol consumption is related to alcohol-related problems later in life. One study found that early onset of alcohol use (i.e., by age 12) was associated with subsequent alcohol abuse and related problem behaviors in later adolescence, including alcohol-related violence, injuries, drinking and driving, absenteeism from school or work, and increased risk for using other drugs (Gruber et al. 1996). Another study found that people who begin drinking before age 15 are 4 times more likely to develop alcohol dependence during their lifetime than are people who begin drinking at age 21 (Grant and Dawson 1997). Therefore, it is clearly an important public health goal to delay the initiation of alcohol use among young adolescents for the benefit of their current and long-term health.

To develop effective programs to prevent alcohol use among young adolescents, it is necessary to first identify the causes of use. The identification of those causes involves a combination of theory and research. According to the theory of triadic influence (TTI), which integrates many behavioral theories into a comprehensive “mega-theory” of health behavior, all behaviors have roots in three domains: the person’s personal characteristics, current social situation, and cultural environment (Flay and Petraitis 1994). The TTI also specifies different levels of influence on behavior for various factors. For example, proximal factors directly pertain to the drinker (e.g., attitudes and perceived norms around alcohol) and more distal factors pertain to the drinker’s environment (e.g., parental practices or laws and policies influencing access to alcohol).

Consistent with the TTI, personal, social, and environmental factors repeatedly have been found to be associated with alcohol use among adolescents (Hawkins et al. 1992; Komro et al. 1997). Personal influences promoting alcohol use include rebelliousness, tolerance of deviance, a high value on independence and nonconformance, low school commitment and achievement, positive beliefs and attitudes toward alcohol use, and lack of self-efficacy to refuse offers of alcohol. Social influences favoring adolescent alcohol use include low socioeconomic status and minimal parental education, family disruption and conflict, weak family bonds, low parental supervision, parental permissiveness and lack of rules about alcohol use, family history of alcoholism, peer alcohol use, perceived adult approval of use, and perceived peer approval of use. Important environmental influences on youth alcohol use include the legal, economic, and physical availability of alcohol as well as cultural norms around use.

This theoretical framework, which is supported by research on risk and protective factors (i.e., etiological research), provides a comprehensive understanding of the factors that influence the onset of alcohol use among adolescents. Furthermore, the framework offers practical guidance on developing strategies to prevent adolescent alcohol use. Indeed, the enhanced understanding of the interrelatedness of personal, social, and environmental factors in determining behavior has influenced prevention efforts considerably. Thus, the focus of prevention approaches has broadened from individual personality characteristics to the social world of the adolescent (e.g., family and peers) and to macrolevel environmental factors (e.g., community and societal messages, norms, and availability) (Perry et al. 1993*a*; Wagenaar and Perry 1994).

As researchers and clinicians develop comprehensive approaches to the prevention of adolescent alcohol use, they must continue to identify the most important characteristics of different intervention strategies that contribute to the strategies’ effectiveness. The following sections and the table summarize current knowledge regarding the most promising components of the whole spectrum of prevention approaches, including school, extracurricular, family, policy, and community strategies.

## School Strategies

The goal of many school-based programs is to reduce the onset and prevalence of adolescent alcohol use by decreasing personal and social risk factors and strengthening personal and social protective factors. Several successful tobacco, alcohol, and marijuana prevention curricula exist, including Life Skills Training (Botvin et al. 1995), Project Northland (Perry et al. 1996), the Midwestern Prevention Project (Pentz et al. 1989), Project SMART (Hansen and Graham 1991), and Project ALERT (Ellickson et al. 1993). These programs have given researchers a better understanding of important components for classroom-based programs. Both meta-analyses (e.g., Tobler 1992; Tobler et al. 2000) and reviews of effective programs (Drug Strategies 1996; Dusenbury and Falco 1995) have identified the following factors as critical components of successful curricula:

- Program development based on behavioral theory and knowledge of risk and protective factors
- Developmentally appropriate information about drugs, including information on the short-term effects and long-term consequences of their use
- The development of personal, social, and resistance skills to help students identify internal pressures (e.g., anxiety and stress) and external pressures (e.g., peer pressure and advertising) to use drugs and to give students the skills to resist these pressures while maintaining friendships
- An emphasis on normative education that reinforces the awareness that most adolescents do not use alcohol, tobacco, or other drugs
- Structured, broad-based skills training, such as goal setting, stress management, communication skills, general social skills, and assertiveness skills
- Interactive teaching techniques, such as role playing, discussions, and small-group activities to promote active student participation
- Multiple sessions over several years, particularly during middle school
- Teacher training and support from program developers or prevention experts
- Active family and community involvement
- Cultural sensitivity—for example, by including activities that require teacher and student input and which can be tailored to the cultural experience of the classroom.

Several studies have compared the effectiveness of different types of school-based programs. For example, two recent meta-analyses compared interactive with noninteractive curricula. Interactive curricula include the components described above, with a substantial amount of time spent in activities that foster the development of interpersonal skills. Noninteractive curricula are more lecture oriented and stress drug knowledge or affective development (i.e., personal insight, self-awareness, and values). The analyses found that interactive curricula were more effective than noninteractive curricula in preventing alcohol, tobacco, and other drug use among youth (Tobler and Stratton 1997; Tobler et al. 2000).

Interactive programs can be further divided into three categories based on their focus on social influences, comprehensive life skills, and system-wide change, respectively. Of these three categories, the system-wide change programs were most effective in preventing overall drug use (including alcohol use), followed by comprehensive life skills and social influences programs (Tobler et al. 2000). System-wide change programs, in turn, are of two types: (1) school-based programs that are actively supported by family and/or community (e.g., Project Northland, which is described below in the section “Multicomponent Strategies”) and (2) programs that provide a supportive school environment but do not involve the family and/or community.

A more recent meta-analysis examined the relative effectiveness of two types of interactive programs—comprehensive life skills programs and social influences programs—and determined specific drug use outcomes for both strategies (Roona et al. in press). In contrast to the findings by Tobler and colleagues (2000), the results indicated that the social influences programs were significantly more effective than the comprehensive life skills programs in reducing alcohol abuse, especially for youth in middle school, where most prevention curricula are implemented. The differences in findings probably stem from the fact that Tobler and colleagues (2000) studied the effects of the programs on overall drug use, whereas the study by Roona and colleagues (in press) was specific to alcohol abuse. Overall, however, the investigators concluded that neither program type significantly reduced alcohol use prevalence and that comprehensive life skills programs actually increased alcohol use. These findings may be explained by the fact that alcohol use is highly ingrained in U.S. culture and is the most difficult type of drug to prevent among adolescents using classroom-based programs.

**Table:** Key Components of Strategies to Prevent Underage Drinking

Type of Strategy	Key Components
School Strategies^a^	Based on behavioral theory and knowledge of risk and protective factors Developmentally appropriate information about alcohol and other drugs Development of personal, social, and resistance skills Emphasis on normative education Structured, broader-based skills training Interactive teaching techniques Multiple sessions over multiple years Teacher training and support Active family and community involvement Cultural sensitivity
Extracurricular Strategies^b^	Supervision by positive adult role models Youth leadership Intensive programs Incorporation of skills building Part of a comprehensive prevention plan
Family Strategies^c^	Improvement of parent-child relations using positive reinforcement, listening and communication skills, and problem solving Provision of consistent discipline and rulemaking Monitoring of children’s activities during adolescence Strengthening of family bonding Development of skills Involvement of child and parents
Policy/Community Strategies^d^	Excise taxes Minimum legal drinking age of 21 Citizen action to reduce commercial and social availability of alcohol

SOURCE:

a Dusenbury and Falco 1995

b Carmona and Stewart 1996

c Ashery et al. 1998; Etz et al. 1998; National Institute on Drug Abuse (NIDA) 1997

d Grossman et al. 1994; Holder et al. 1997; Lockhart et al. 1993; Perry et al. in press; Wagenaar et al. 2000a,b; Wagenaar and Toomey 2000

The study by Roona and colleagues (in press) included only results on program effectiveness over the first year after the intervention. It is also important, however, to consider more long-term results when analyzing the effectiveness of prevention programs. Such long-term analyses have been conducted for several programs, demonstrating that some result in long-term reductions of tobacco and marijuana use, but not alcohol use, among adolescents (Ellickson et al. 1993; Pentz et al. 1989; Johnson et al. 1990). This finding again supports the greater resistance of alcohol use behavior to change.

The sole curricula-only prevention program that has reported long-term effects on alcohol use is Life Skills Training (Botvin et al. 1990, 1995). This program consists of 3 years of prevention curricula for middle or junior-high school students and includes 15 sessions during the first year, 10 sessions during the second year, and 5 sessions during the third year. The curricula cover drug information, drug-resistance skills, self-management skills, and general social skills. A long-term followup study indicated that this program had long-term effects on tobacco, alcohol, and marijuana use through grade 12 (Botvin et al. 1995); however, no alcohol results were reported in the article presenting results from 1 year past high school (Botvin et al. 2000).

The Life Skills Training curricula focus on changes only at the individual level. A recent etiological analysis, however, indicates that individual-level variables only account for a small percentage of the variance in alcohol use among adolescents (Griffin et al. 2000). Accordingly, Griffin and colleagues (2000) concluded that classroom-based prevention efforts should be complemented with family, community, and policy initiatives that facilitate change in the larger social environment. Such approaches are reviewed in the following sections.

## Extracurricular Strategies

About 40 percent of adolescents’ waking hours are discretionary—not committed to such activities as eating, school, homework, chores, or working for pay—and many young adolescents spend virtually all of this time without companionship or supervision by responsible adults (Carnegie Council on Adolescent Development 1992). Discretionary time outside of school represents an enormous potential for either desirable or undesirable behaviors, such as alcohol and other drug use. Several studies have found that young adolescents who are more likely to be without adult supervision after school have significantly higher rates of alcohol, tobacco, and marijuana use than do adolescents receiving more adult supervision (Mulhall et al. 1996; Richardson et al. 1993).

Scales and Leffert (1999) conducted a comprehensive literature review on the effects of involvement in youth programs (e.g., sports, recreation, camps, mentoring, and drop-in centers) on adolescent development. They found that involvement in youth programs is associated with the following outcomes:

- Better development of life skills
- Greater communication skills
- Fewer psychosocial problems
- Decreased involvement in risky behaviors, such as drug use
- Decreased juvenile delinquency and violence
- Decreased risk of dropping out of school
- Increased academic achievement
- Increased safety.

Another study also found involvement in extracurricular activities to be related significantly to reduced adolescent alcohol, tobacco, marijuana, and other drug use (Jenkins 1996). Widely cited meta-analyses (e.g., Tobler 1992) compared the effectiveness of two types of extracurricular programs: peer programs and alternative programs. Peer programs were defined as interventions that included social and life skills training, including refusal skills. Alternative programs were defined as interventions that included the provision of positive activities more appealing than drug use (e.g., sports activities). The meta-analyses found that alternative programs overall were less effective than peer programs. Among the alternative programs, those that involved high-risk youth and that involved many hours of activities were most effective.

Similar findings were reported in a review of alternative programs published by the Center for Substance Abuse Prevention (CSAP) (Carmona and Stewart 1996). That report concluded that there was no strong research support for the alternative approach. The review offered the following conclusions based on the available research:

- Alternative approaches seem to be most effective with high-risk youth who may not have adequate adult supervision and a variety of activities available to them in their daily life.
- Youth involvement in the planning and implementation of alternatives may enhance participation and effectiveness.
- More intensive programs seem to be most effective.
- Alternative programs should incorporate skills-building components into their design.
- Alternative programs should be one part of a comprehensive prevention plan serving to establish strong community norms against alcohol use.

As noted by Carmona and Stewart (1996), an important component of extracurricular activities appears to be active youth leadership. This conclusion was supported by a study by Komro and colleagues (1996), who reported that youth who participated in planning alcohol-free activities for their peers significantly reduced their alcohol use. However, more research using rigorous controlled designs is needed to understand the effects of involvement in extracurricular activities and youth leadership on early onset of alcohol use.

## Family Strategies

Several sources have recommended family involvement as important for the success of alcohol prevention strategies (Drug Strategies 1996; Dusenbury and Falco 1995; National Institute on Drug Abuse [NIDA] 1997). Family factors, such as parent-child relationships, discipline methods, communication, monitoring and supervision, and parental involvement, can significantly influence alcohol use among youth (Bry et al. 1998). Because of increasing demands on their time and attention, however, parents are spending less time with their children and therefore need strategies and ideas to help them effectively parent their children (Kumpfer 2000).

Promising family strategies for preventing alcohol, tobacco, and other drug use include structured, home-based parent-child activities; family skills training; behavioral parent training; and behavioral family therapy. Reviews of family skills training indicate that enhancement of the following parenting skills is important for the prevention of alcohol use (Ashery et al. 1998; NIDA 1997):

- Improving parent-child relations by using positive reinforcement, listening and communication skills, and problem solving
- Providing consistent discipline and rulemaking
- Monitoring children’s activities during adolescence
- Strengthening family bonding.

Various studies have identified several components that contribute to the success of family based prevention interventions. One major component is a focus on skill development rather than on simple education about appropriate parenting practices (Etz et al. 1998). Another important component is the involvement of both parents and children in individual and group training sessions (Etz et al. 1998). Several studies have found that parent and family training programs both improve parenting skills and reduce problem behaviors among children (Ashery et al. 1998; NIDA 1997).

Examples of successful parenting programs include the Preparing for the Drug-Free Years (PDFY) program and the Iowa Strengthening Families Program (ISFP) (Kumpfer et al. 1996; NIDA 1997; Spoth et al. 1999a,b). The PDFY program consists of five competency-training sessions for parents, with young adolescents attending one of those sessions together with their parents. The ISFP comprises seven sessions, each attended jointly by youth and their parents. Comparisons of both interventions with control families found positive effects on parents’ child management practices and parent-child relations, improved youth resistance to peer pressure toward alcohol use, reduced affiliation with antisocial peers, reduced levels of problem behaviors, and delayed substance use initiation (Kumpfer et al. 1996; Spoth et al. 1999a,b).

A less intense family involvement approach is based on including parents in homework assignments around issues of alcohol use, thereby increasing the likelihood that alcohol, tobacco, and other drug use is discussed at home, and potentially enhancing parenting skills by increasing communication between parent and child and providing behavioral tips to parents. For example, Project Northland, which is described later in this article, used homework assignments to engage families and provide behavioral tips.

## Policy Strategies

Adolescent alcohol use also is determined by important environmental influences, such as the legal, economic, physical, and social availability of alcohol (Wagenaar and Perry 1994). Accordingly, lawmakers have implemented several policy strategies targeting these influences to reduce the availability of alcohol to youth. These strategies include raising the minimum legal drinking age (MLDA), curtailing commercial access, limiting social access, and reducing economic availability.

### Increasing the MLDA

The effectiveness of alcohol policies in significantly reducing alcohol-related problems has been well demonstrated by changes in the MLDA and the resulting consequences. During the early 1970s, 29 States lowered their MLDA, typically from age 21 to ages 18, 19, or 20. As concern about increasing rates of alcohol-related traffic crashes among young people grew, however, a grassroots movement developed in many States, putting pressure on State governments to raise the MLDA back to age 21. In 1984, the Federal government passed the Uniform Drinking Age Act, which provided for a reduction in Federal funds to States that did not raise their MLDA to age 21, and by 1988, all States again had a MLDA of 21.

The MLDA is the most-studied alcohol policy, with 132 published studies (Wagenaar and Toomey 2001). Included in these are well-controlled investigations providing clear evidence that a higher MLDA can effectively reduce drinking as well as alcohol-related car crashes and other injuries among teenagers.

Though effective, the increase in MLDA to age 21 has had only modest enforcement^1^ (Wagenaar and Wolfson 1994). For example, youth report that they have easy access to alcohol from both licensed establishments and social sources (e.g., friends or acquaintances) (Wagenaar et al. 1996). These reports are substantiated by purchase-attempt studies, which directly test the propensity of establishments to sell alcohol to youth without requiring identification. In the early 1990s, such studies found that young buyers could purchase alcohol with no age identification in approximately 50 percent of the purchase attempts (Forster et al. 1995). In addition, youth frequently receive alcohol from social providers, including parents, friends, coworkers, and even strangers (Wagenaar et al. 1996). Accordingly, public health professionals and activists in many communities are working to reduce youth access to alcohol from both commercial and social providers using public and institutional policy changes, such as the ones described in the following sections.

### Policies to Reduce Commercial Access

To address the problem of alcohol availability from commercial providers, communities have conducted enforcement campaigns using compliance checks. During these checks, law enforcement officers supervise attempts by underage youth to purchase alcohol from licensed establishments. When an illegal sale is made, penalties are applied to the license holder and/or the clerk or server who made the sale. Such compliance checks can significantly reduce sales to minors (Preusser et al. 1994; Grube 1997). State and local laws providing for graduated administrative (as opposed to criminal) fine and license suspension penalties for establishments that sell to minors may improve the effectiveness of these enforcement efforts because the increased certainty of penalties is a key component of deterrence-based approaches (Ross 1992).

Other policy tools to reduce youth access to alcohol from commercial sources include requiring servers of alcohol to be trained to detect false age identification, designing drivers’ licenses to clearly indicate whether someone is underage, and banning or regulating home deliveries of alcohol. Studies evaluating server-training programs show that such programs by themselves are unlikely to reduce sales to underage youth (Howard-Pitney et al. 1991; Toomey et al. 2001). Training programs may be useful, however, for creating a political climate that decreases resistance to enforcement campaigns that can effectively reduce sales to minors.

Home deliveries of alcohol may make it even easier for youth to obtain alcohol from a retail establishment because the transaction occurs in completely unmonitored settings. Approximately one-half of the States in the United States allow alcohol delivery from retail establishments to private residences. The only published study of teen use of home delivery found that 10 percent of the 12^th^ graders and 7 percent of the 18- to 20-year-olds reported consuming home-delivered alcohol (Fletcher et al. 2000). A limitation of this study is that it did not ask whether it was the underage youth or an adult who had ordered the delivery of alcohol.

Recently, State and national policy-makers have proposed restrictions on home delivery of alcohol ordered from Internet sites. Although debates over these controversial proposals involve apparent concern for reducing youth access to alcohol, home delivery from local retail outlets is a more likely source of alcohol than Internet orders, at least in part because it provides more immediate access to alcohol. Internet sales require youth to plan weeks in advance to purchase alcohol for a drinking event, require a credit card, involve careful planning when and where the alcohol will be delivered, and potentially require storage until the drinking event occurs. Restrictions on retail home deliveries of alcohol, however, are not included in the policy debates on Internet sales; therefore, it appears that policy attention to alcohol Internet sales may have more to do with the varying economic interests of local versus national alcohol distributors and retailers. The effects of restrictions on Internet or retail home deliveries on youth alcohol use have not been studied.

### Policies to Reduce Social Access

Policy tools for limiting youth access to alcohol from social providers attempt to reduce the frequencies of underage drinking parties and of adults illegally providing alcohol to youth. Some of these prevention approaches are being implemented at the community level. For example, communities may address underage drinking parties by creating enforcement mechanisms, such as noisy assembly ordinances, that allow law enforcement officers to enter private residences where underage drinking is occurring.^2^ Communities can also require beer kegs to be registered at the time of retail sale. Using a keg’s unique identification number and the registration information, police officers can identify and penalize adult purchasers of kegs used at parties where underage guests are caught drinking. To deter adults from illegally giving alcohol to youth, some States have enacted social host laws that allow third parties to sue social providers when provision of alcohol to youth results in a death or injury. Although many possible policy strategies have been identified that may help reduce social access to alcohol, little research has been done to evaluate the specific effects of these strategies.

### Policies to Reduce Economic Availability

Policies also can help reduce the economic availability of alcohol. A large number of econometric studies have clearly demonstrated an inverse relationship between price and consumption of alcohol—that is, higher prices result in reduced consumption. (For more information on the effects of price on alcohol consumption, see the article in this issue by Chaloupka and colleagues, pp. 22–34.) Policy simulation studies suggest that this relationship exists among the general population as well as among adolescents. Thus, higher alcohol prices may substantially reduce both the frequency and the amount of teen drinking, even among youth who are already heavy alcohol consumers (Laixuthai and Chaloupka 1993). In fact, price increases may be particularly effective in reducing youth drinking, because heavy drinkers in young populations are more affected by price than are heavy drinkers in the general population (Godfrey 1997; Chaloupka and Wechsler 1996).

One policy that has been used to raise the price of alcohol is to increase the excise tax on alcohol. Although alcohol excise taxes are often raised for revenue-generating reasons, several studies suggest that higher excise taxes may affect youth consumption and its consequences. Higher taxes on alcohol are associated with less drinking among 16- to 21-year olds (Grossman et al. 1994) and high school students (Lockhart et al. 1993). Higher taxes are also associated with fewer traffic fatalities among youth (Saffer and Grossman 1987), higher graduation rates from college (Cook and Moore 1993), and less violence among college students.

## Community Strategies

Community participation is critical for creating comprehensive changes in institutional policies (e.g., of alcohol establishments, media outlets, and schools) and public policies aimed at reducing youth access to alcohol. Several community trials have included community-organizing components to mobilize and successfully change policies addressing public health issues (Wagenaar et al. 2000*a*; Holder et al. 1997).

Only one community trial—Communities Mobilizing for Change on Alcohol (CMCA)—has focused solely on policy changes to reduce youth access to commercial and social sources of alcohol. CMCA tested a community-organizing intervention in a trial involving 15 communities that were randomly assigned to receive the intervention or to serve as control communities. The goal of the community-organizing intervention was to reduce the accessibility of alcoholic beverages to youth under age 21. Through the organizing effort, diverse groups of people across the intervention communities developed and implemented strategic action plans to influence a wide array of institutional policies (Wagenaar et al. 1999). The intervention was successful in several respects. For example, it changed alcohol merchant practices around selling to underage youth and reduced the propensity of 18- to 20-year olds to buy alcohol in a bar, provide alcohol to other teens, or consume alcohol (Wagenaar et al. 2000*a*). Furthermore, following the intervention, arrests for driving under the influence among 18- to 20-year olds were significantly lower in the intervention communities than in the control communities (Wagenaar et al. 2000*b*).

Two other community trials—the Community Trials Project (CTP) and the Saving Lives Program—have also addressed underage drinking, although the focus of these studies expanded beyond the underage population. The goal of the CTP was to reduce injury and deaths related to alcohol use among all age groups (Holder et al. 1997). The intervention included the following components:

- Involvement of the media to increase awareness
- Training of alcohol-retail establishments, including information on preventing sales to underage patrons
- Compliance checks conducted by law enforcement to reduce illegal alcohol sales to underage patrons
- Increased enforcement of drunk-driving laws
- Reduction of alcohol availability through regulation of alcohol outlets.

Following the intervention, sales rates to buyers who appeared to be under age 21 were lower in the three intervention communities than in the three comparison communities (Grube 1997). The intervention communities also showed reductions in self-reported drinking-and-driving rates, nighttime injury crashes, alcohol-related crashes, and assault injuries among the general population (Holder et al. 2000).

The Saving Lives program, which was conducted in six communities in Massachusetts, also involved community mobilization to address drinking and driving among all age groups (Hingson et al. 1996). The intervention included multiple strategies that addressed alcohol-impaired driving as well as other traffic problems, such as speeding, other moving violations, and seat belt use. Following the intervention, the relative decrease in alcohol-involved fatal traffic crashes was 42 percent in the intervention communities compared with the rest of the State (the absolute change was from 69 crashes to 36 crashes in the intervention communities). Furthermore, self-reported drinking-and-driving among 16- to 19-year-olds was reduced by 40 percent in the intervention communities compared with the rest of Massachusetts.

## Multicomponent Strategies

Although various individual strategies have been successful in preventing youth alcohol use, a more comprehensive approach combining several of the intervention strategies described above might be even more effective. Two studies—the Midwestern Prevention Project and Project Northland—have combined school, family, and community strategies to prevent alcohol use among adolescents; their results are described in the following sections.

### Midwestern Prevention Project

The Midwestern Prevention Project, which was not specific to alcohol use but addressed all types of drug use, consisted of the following four components:

- A 10-session school program emphasizing drug-use-resistance skills training, delivered in grade 6 or 7; this component also included homework sessions involving active interviews and role plays with parents and family members
- A parent organizations program for reviewing school prevention policy and training parents in positive parent-child communication skills
- Initial training of community leaders in the organization of a drug abuse prevention task force
- Mass media coverage of the program.

The study was composed of eight representative Kansas City communities that were randomly assigned either to the full program including all four components or to a control program including only the community organization and mass media components. After 3 years, students in the communities implementing the full program had lower rates of tobacco and marijuana use, but not alcohol use; this follows the previously described findings that alcohol use patterns appear to be the most difficult to change.

### Project Northland

Project Northland was designed to prevent or reduce alcohol use among young adolescents using a comprehensive, multicomponent intervention that targeted both the supply of and demand for alcohol. Project Northland was evaluated using 20 school districts from northeastern Minnesota that were randomly assigned either to the treatment or control condition. The students participating in the study were surveyed from grades 6 through 12. The intervention was conducted in three stages: a first intervention phase, an interim phase, and a second intervention phase. The first intervention phase, which was conducted when the students were in grades six through eight, included: (1) social behavioral curricula, (2) peer leadership and extracurricular social opportunities, (3) parental involvement and education, and (4) community-wide task forces (Perry et al. 1993*b*). At the end of 3 years, a smaller percentage of students in the intervention communities reported drinking or beginning to drink compared with students in the control communities. Furthermore, among students in all districts who at the beginning of sixth grade reported never having consumed alcohol, those in the intervention communities were not only less likely to drink 3 years later but also had lower rates of cigarette and marijuana use (Perry et al. 1996).

The interim phase of the study occurred when the students were in grades 9 and 10. During those years, only minimal intervention (i.e., a five-session classroom program) took place, and drinking rates between the treatment and control groups began to converge. In fact, by the end of grade 10, no significant differences existed between the two groups (Williams and Perry 1998).

In the second intervention phase, when the students were in grades 11 and 12, they were exposed to various interventions, including an 11^th^ grade classroom curriculum, parent postcards, mass media involvement, youth development activities, and community organizing (Perry et al. 2000). As a result of the intensified intervention, the alcohol use patterns of the treatment and control groups began to diverge again by the end of the 11^th^ grade, and the differences between groups were marginally significant for those students who had not used alcohol at the beginning of 6^th^ grade (Williams et al. 1999).

An analysis comparing the trajectories of alcohol use between the treatment and control groups (i.e., a growth curve analysis) was conducted for all three phases of Project Northland. During the first intervention phase, the increase in alcohol use was significantly greater in the control group than in the intervention group. Conversely, the increase in alcohol use was significantly greater in the intervention group than in the control group during the interim phase, when there were minimal program efforts. Thus, the students in the intervention group seemed to return to the level of drinking that was normative in their communities. Fortunately, that trend was reversed again during the second intervention phase. During that period, the increase in alcohol use was again greater in the control group than in the intervention group (*p*<0.02), demonstrating the positive and significant impact of the second intervention phase (Perry et al. in press). In addition, the community-organizing intervention component during the second intervention phase, which focused on community action team-initiated compliance checks of alcohol outlets, successfully reduced the ability of youthful-appearing 21-year-olds to purchase alcohol without age identification (*p*=0.05) (Perry et al. in press).

## Conclusion

Adolescent alcohol use is one of the most difficult behaviors to change because alcohol use is so ingrained in the U.S. culture. Adolescents choose to consume alcohol, not just because of personal characteristics, such as personality type or level of social skills, but also because it is a part of daily life in their communities and, for many youth, in their homes (Wagenaar and Perry 1994). As Wagenaar and Perry indicate in their theoretical model (1994), numerous social and environmental influences affect adolescents, including messages they receive from advertisements, community practices, adults, and friends about alcohol. Comprehensive interventions targeting underage drinking may need to counter or change all of these messages to motivate individual adolescents to choose not to consume alcohol.

Researchers’ knowledge about effective interventions to reduce underage drinking—particularly about school-based programs targeting individual-level factors—has grown substantially during the past decade, and investigators have identified key components of state-of-the-art school-based programs. By themselves, however, these programs are unlikely to create sustained reductions in underage drinking. Instead, school-based programs may need to be combined with extracurricular, family, and policy strategies that help change the overall social and cultural environment in which young people live to create sustained decreases in consumption and alcohol-related problems among youth.

Although key components of non-school-based strategies have been identified, further research is needed in many of these areas to understand fully what factors must be targeted and what methods can best achieve those targets and reduce underage drinking. As researchers, clinicians, and policymakers learn more about each strategy, they need to synthesize this knowledge to develop multicomponent projects consisting of high-quality and complementary components that together create interventions strong enough to overcome the drinking culture found throughout U.S. communities.

## Footnotes

1 The little enforcement that occurred in the late 1980s and early 1990s primarily involved citing underage drinkers rather than the adults who were illegally selling or providing alcohol to underage youth.

2 An example of such an ordinance can be found on the Internet at www.epi.umn.edu/alcohol.
